# The Importance of the Veteran’s Voice: Three Veterans Recount Their Engagement with VA Research: Importance of Veteran Voice

**DOI:** 10.1007/s11606-021-07103-x

**Published:** 2022-03-29

**Authors:** Harry L. Maxwell, Paula A. Smith-Benson, Beverly Velasquez, Dana S. Kaminstein, Kimberly M. Brown

**Affiliations:** 1 Center for Health Equity Research and Promotion, Crescenz VA Medical Center, 3900 Woodland Ave., Philadelphia, PA 19104 USA; 2 Organizational Dynamics, School of Arts & Sciences, University of Pennsylvania, Philadelphia, PA USA

**Keywords:** patient engagement, Veteran, empowerment, outcomes, Veterans Affairs, health service research

In the effort to join scientifically important outcomes with patient-important outcomes, Veterans are making a significant contribution to health services research.^1^ In a prior publication, we describe Veteran engagement at the VA Center for Health Equity Research and Promotion (CHERP) in detail, including nuances and subtleties of the relationship.^2^ To summarize, the Veterans Community Advisory Board (VCAB), an independent, active and empowered Board within a federal agency, advocates for the Veteran perspective in VA research.^1,3^ The members personify the vulnerable communities at the heart of CHERP’s health equity research mission: e.g., Veterans with stigmatizing mental, social, and health conditions (i.e., addiction, homelessness, HIV+, etc.), Veterans over 65, LGBTQ+ Veterans, women Veterans, and Veterans of color. During a two-and-a-half-hour monthly meeting, a variety of topics are discussed and feedback given to investigators. The VCAB has also designed and instituted enhancements in the ways that Veterans participate in and learn about research.

To portray what we mean by active and empowered, and in response to a call for papers, we asked VCAB members if they would like to contribute to an article. Three members volunteered to write open-ended reflections about what was important to them regarding Veteran engagement in research: Board Chair Harry “Chuck” Maxwell, Board Vice Chair Paula Smith-Benson, DMgt, MSN, RN, and Beverly Velasquez, LCSW. To ensure we captured the Veterans’ own words—the Veterans’ voice—and the meanings that the Veterans wanted to convey, we utilized an inductive, socially constructed, modified content analysis. Six themes emerged: (1) Formation, their initial reactions to the way their involvement shaped the Board; (2) Diversity and leadership; (3) Veteran voices and feelings of empowerment; (4) Training helped members feel confident in speaking to researchers; (5) Personal and professional growth; and (6) Accomplishments.

## FORMATION

Initially, the VCAB lacked form and structure. Through a focus group, referrals, and various outreach efforts, the Board attracted a diverse membership.

BV: The VCAB was introduced to me by a former Social Work intern, who was also a researcher at CHERP. He could see how strong of an advocate I could be for Veterans. He really was encouraging to me during a time when I needed a challenge. I found it exciting to think that I could be involved in creating this board.

HCM: When first approached my thoughts were, “Me?” I knew nothing about research and immediately thought “No.” I pondered what this could look like and thought of other Veterans that I could suggest for membership. I am proud to say, after four years, they are still current and engaged members.

PSB: My partner was first approached but she felt I would be a better fit because I was already passionate about advocacy for Veterans and about VA Research. Being on the VCAB was emancipatory, because I was a part of a group who had been silenced and oppressed. Being a part of the VCAB enabled me to use my voice.

## DIVERSITY AND LEADERSHIP

The Diversity and Leadership of the Board was also a concern for the members.

PSB: As a black LGBTQ+ female Veteran with a history of homelessness, stigmatized mental health conditions, and military sexual trauma, I serve as a proxy to represent the multiple angles in which these research populations see the world.

HCM: I wanted to be a leader in this new entity because I had the networks to make things happen. The fact that I was an African American, an Air Force Veteran, and an employee made me feel that the Board was truly diverse.

## IMPORTANCE OF THE VETERAN’S VOICE

All VCAB members are directed to explore their readiness to speak their mind about difficult issues and uncomfortable topics. Over time, the confidence their voices will be heard enabled feelings of influence and empowerment to take shape.

BV: I think the VCAB has a great deal of power and shows that Veterans can come together as a diverse group. What is most important is the Veteran’s voice.

PSB: I see the VCAB as an opportunity to provide voice and empowerment for these unique populations. However, incorporating my voice alone is only a preliminary element. Including them as researchers, co-researchers, and decision-makers across all levels of the Veteran stakeholder system is my objective.

HCM: I thought this could be an opportunity to make sure the Veteran’s voice is represented and considered in the conversation about research. Through CHERP trainings and interactions with Principal Investigators, I cannot overstate how Veteran empowerment levels the field.

## TRAINING

Training in VA research priorities and techniques helped VCAB members blossom. These educational requirements are the result of a desire to grant members a federal appointment that enables them to hear detailed information about patient care. The training members receive is designed to provide an overview of VA research. Initially, every member completes online CITI training modules. Next, members are given a 120-minute immersion into VA research fundamentals entitled "Research 101." Furthermore, members receive in-service training about our VA facility, and on-going annual training requirements.

HCM: The training regarding research was essential to understanding and communicating with researchers. As a photographer, I attended a CHERP Health Equity conference several years ago. Before that, I had no idea of how much research CHERP did and how far their tentacles reached. This is where I was recruited to become a member.

BV: In my work with this board I learned how research is introduced to the medical center, how important research is to our future. I never cared for research when I was in school. This is different, because I have the opportunity to review projects that come to the medical center. Learning the history of VA Research was very interesting to me.

## PERSONAL AND PROFESSIONAL GROWTH

Personal and professional development was an unexpected benefit to being involved with the VCAB. Members built confidence on account of the variety of ways to be a member: committee membership, public outreach, public speaking, Veteran directed research projects, and engagement with national VA leaders. Expressing their ideas created a virtuous cycle leading to greater willingness to act on those ideas.

PSB: I experienced growth transitioning into a leadership position in the VCAB; it has been both empowering and inspiring. The work with the VCAB has influenced my dissertation which is focused not only on leadership but also health equity. The VCAB has changed my postdoctoral trajectory towards becoming a health equity researcher.

BV: The VCAB has shaped me as a social worker by making me aware of disparities in healthcare. I have also shaped the VCAB as a social worker by giving a social work perspective on these issues. I was asked by a supervisor, out of all of the work that I have done, what was my greatest achievement? What was surprising to me was that the first thing that came to mind was the VCAB.

## ACCOMPLISHMENTS

Participation in the VCAB lends a status and authority that many members had not enjoyed previously.

BV: The growth of this board has been inspiring and we have shown ourselves to be quite valuable to the medical center.

PSB: A memorable experience of mine was when a principal investigator came to the VCAB to request assistance with recruiting African American men for a study. The VCAB recommended that we observe the Informed Consent and offer suggestions to make it Veteran-centered. I recognized that mistrust from black individuals is a result of the complex intersection of historical atrocities. I offered suggestions on images, wording, and tone to tailor the materials to the potential participants. The VCAB is spearheading an effort to rebuild trust and improve the well-being of all Veterans.

HCM: It was natural for me to want to share this good news with other Veterans, so I designed a video, posters, an information brochure as well as public outreach. Additionally, we originated the first-ever Veterans Research Town Hall to educate, address Veteran concerns and dispel myths about research. An exit survey completed by over 125 attendees confirmed that as a result of the town hall they would be more apt to participate in VA research studies.

## CONCLUSION

Above, we have illustrated that membership in the Veterans Community Advisory Board leads to a number of protective factors for Veterans. Having a say in the formation of the board meant that the resulting community would mirror the needs of its members. Training eased the discomfort with complex research and their ability to work together as a group. Personally, and professionally, members’ confidence grew to tackle a broad range of complicated issues around how Veterans get their care. The partner authors of this piece, who are also researchers, recognize the unique perspectives and accomplishments of our VCAB members. The most important outcome is that members are using their voice, training, and professional confidence to be vocal advocates for Veterans, especially those from vulnerable populations.
